# Coparenting and Parental Involvement During School Transition Among Chinese Mothers and Fathers: Children’s School Liking as a Moderator

**DOI:** 10.3389/fpsyg.2021.769416

**Published:** 2021-11-29

**Authors:** Sisi Tao, Eva Yi Hung Lau

**Affiliations:** ^1^Centre for Information Technology in Education, Faculty of Education, The University of Hong Kong, Pokfulam, Hong Kong SAR, China; ^2^Department of Early Childhood Education, The Education University of Hong Kong, Hong Kong, Hong Kong SAR, China

**Keywords:** coparenting, parental involvement, school liking, Chinese parents, school transition

## Abstract

Parental involvement is a vital social resource that helps children to deal with different challenges in their learning and development in the transition period and may be a strong determinant of children’s outcomes. While the role of fathers has been increasingly recognized, there has been a lack of studies examining the predictive role of mother and fathers’ coparenting to parental involvement and child readiness outcomes. The purpose of this study is to examine the longitudinal association between coparenting behavior and parental involvement for parents with children in the transition to primary school in a Chinese context, and test whether children’s school liking moderated these associations. Using stratified random sampling, 324 children (*M*_age_=70.57months, female=51%) and their parents from 10 kindergartens in Hong Kong participated in the study. Both mothers and fathers provided information about their spouse’s coparenting behavior at Time 1 (the final year of kindergarten), and their parental involvement at home and school at Time 1 and 2 (the first year of primary school). Children’s school liking was assessed by puppet interview at Time 1. Results indicated that maternal cooperation was positively associated with paternal involvement at home and in school, and paternal cooperation was positively associated with maternal involvement at home. Children’s school liking moderated the longitudinal associations between coparenting behavior (Time 1) and parental involvement (Time 2). Specifically, mothers of children with high levels of school liking were involved more in school when they perceived more cooperation from the spouse. Fathers of children with low levels of school liking were less involved in school when they perceived more cooperation, while involved more at home and in school when they perceived more triangulation from the spouse. Additionally, fathers perceiving more triangulation decreased their home involvement when the child reported high levels of school liking. Findings of this study revealed that coparenting, children’s school liking, and parental gender might be important to understanding parental involvement during school transition.

## Introduction

Parental involvement is defined as a multi-faceted concept that includes a wide range of parental practices that take place both at home and in school to aid the development of children (Epstein, 1995; Fan and Chen, 2001). According to social support theory, parental involvement is a vital social resource that helps children deal with different challenges in their development process and may be a strong determinant of children’s outcomes (Cohen and Wills, 1985). Although individual level (e.g., parents’ education level) and school level factors (e.g., teacher invitations) were found to predict parental involvement, less is known about how family-level factors such as coparenting influence parental involvement during the transition to primary school. The goal of this study is to examine the longitudinal relations between coparenting and parental involvement in school transition and to analyze children’s school liking as moderator in these relations.

### Parental Involvement During School Transition

Transition to primary school represents a major challenge for young children (Conn-Powers et al., 1990). Parental involvement is considered to be crucial in helping children cope with different challenges in their school transition (Fan and Chen, 2001). When parents and teachers collaborate to support children, children are more likely to experience a successful school transition and show enhanced school adjustment, generally defined in terms of academic performance (e.g., language and cognitive skills) and school engagement (e.g., school liking; Birch and Ladd, 1997). The positive influence of parental involvement, such as co-reading at home and parental communication with the school has been confirmed as they collectively enhance parent-child relationships and improve children’s school readiness (Li and Rao, 2000; Boonk et al., 2018).

Generally, examples of parental involvement at home include having conversations and collaborating in learning and leisure activities with children, whereas examples of parental involvement in school include communicating with teachers and participating in school events (Epstein, 1995; Fan and Chen, 2001). To better conceptualize parental involvement during the transition to primary school in the Chinese context, Lau et al. (2011) specified home involvement into four subdimensions, including parent instruction (activities that promote children’s self-care and social and emotional skills), parent discussion (discussion of issues related to school), language and cognitive activities (home learning activities that aid children’s language and cognitive skills), and homework involvement (supervision of and assistance with children’s completion of homework). The school involvement was conceptualized into two subdimensions, including home-school conferencing (parents’ school-based involvement in communicating with the school) and school involvement (participation in various school activities to assist the schools’ functioning). Both home and school involvement was found to have a positive influence on children’s school readiness (for a review, see Boonk et al., 2018). As such, it is important to explore factors that predict parental involvement, particularly during the critical transition from kindergarten to primary school.

### Coparenting and Parental Involvement

Coparenting is defined as the way parents coordinate their shared responsibility to rear the child (McHale et al., 2002). Although the conceptualization of coparenting differs in previous theoretical reviews and empirical studies (Margolin et al., 2001; Feinberg, 2003), supportive coparenting and conflicted coparenting are among the most widely used operationalizations of coparenting in studies of parent-child relationships. Supportive coparenting describes how parents value and respect each other through cooperation; while conflicted coparenting refers to how parents intrude upon or exclude the other parent through triangulation and conflict. According to Family Systems theory, both supportive and conflicted coparenting are closely associated with parental involvement and subsequent child outcomes. Specifically, family is a social system with members interdependently influencing each other and developing behavior patterns that are maintained over time (Minuchin, 1985). The mood, or behaviors from one subsystem, e.g., co-parenting, can be transferred to another, e.g., parental involvement to the child (Erel and Burman, 1995). The spillover hypothesis suggests that a couple’s interaction may spill over into parent-child interactions through parents’ moods or behaviors (Krishnakumar and Buehler, 2000). Parents’ conflict of their shared parenting responsibility (i.e., conflicted coparenting) may thus be transferred to parent-child interaction and decreases their parental involvement. Alternatively, the compensatory hypothesis proposes that parental relationship problems may lead to more attention, dedication, and investment from parents toward their child (Kouros et al., 2014). This hypothesis would predict higher levels of parental involvement among couples with low supportive coparenting, given that parents are motivated to invest more time in the parent-child relationship to achieve any unmet needs of love and support in the mother-father relationship.

To date, empirical studies have mostly documented the evidence of the spillover hypothesis but there has been little evidence to support compensatory effects (Hammer et al., 2005; Nelson et al., 2009). For example, consistent with the spillover hypothesis, higher levels of supportive coparenting were associated with higher levels involvement for both mothers and fathers (Schoppe-Sullivan et al., 2008; Hohmann-Marriott, 2011; Berryhill, 2017). Conflicted coparenting was negatively associated with paternal involvement in caregiving and play (Buckley and Schoppe-Sullivan, 2010; Jia and Schoppe-Sullivan, 2011). Although studies demonstrate the associations of coparenting with parental involvement, the results vary depending on which aspects of parental involvement were investigated. It has been suggested to pay special attention to the different dimensions of parental involvement and that they be measured separately (Fan and Chen, 2001). However, research on parental involvement has been fragmented, addressing a range of variables, mostly home-based involvement such as play and caregiving (Buckley and Schoppe-Sullivan, 2010; Jia and Schoppe-Sullivan, 2011) and physical care and cognitive stimulation (Fagan and Cabrera, 2012). More studies using the multidimensional framework of parental involvement (i.e., both at home and in school) are warranted. Additionally, given the cross-sectional nature of most of the above-mentioned studies (e.g., Hohmann-Marriott, 2011), longitudinal investigation addressing the causal effects of coparenting and parental involvement is needed.

### Child School Liking as Moderator

**Figure 1:**
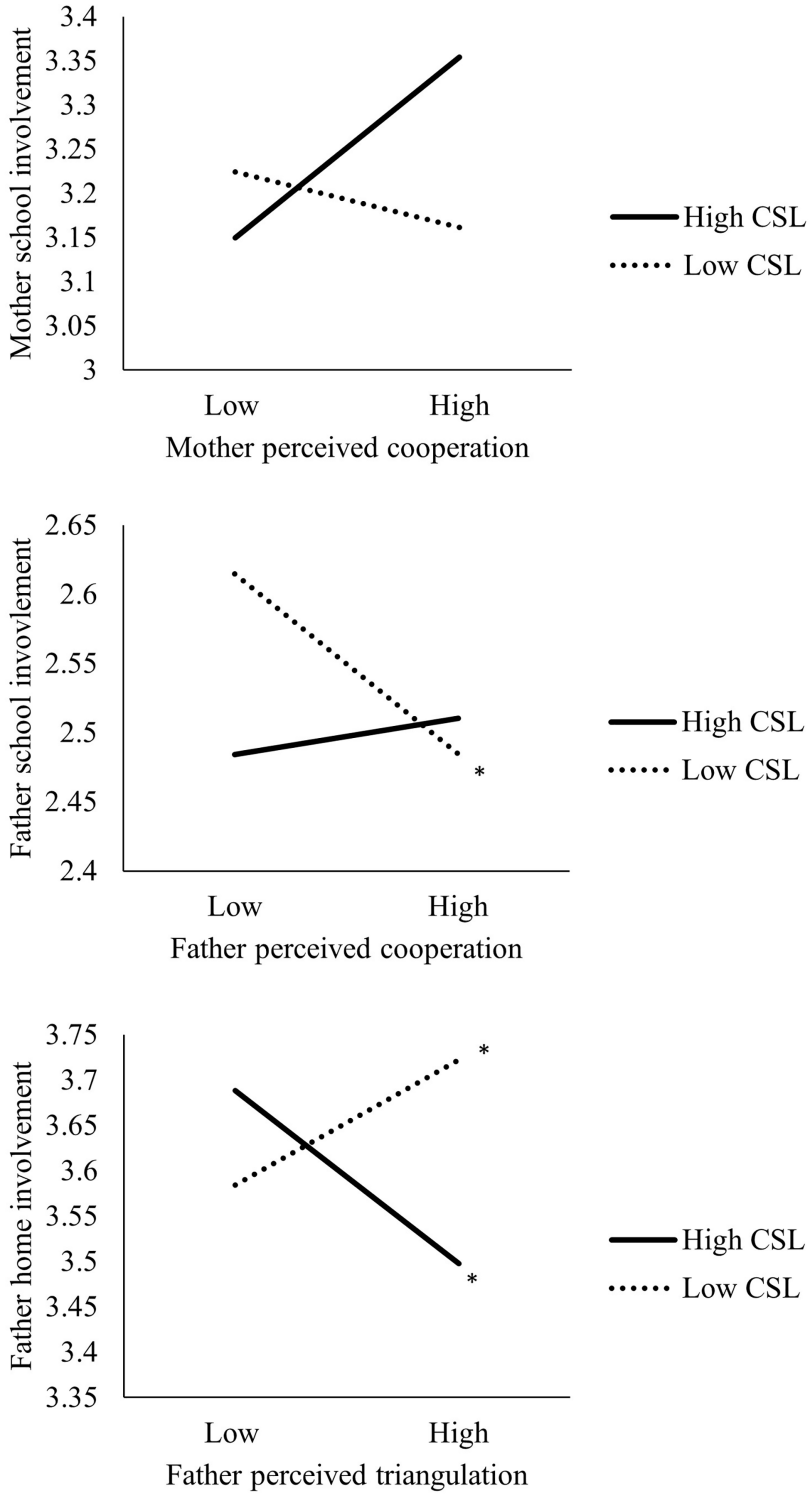
Moderation plots. ^*^p < 0.05.

Since the family is a social system with members interdependently influencing each other over time (Minuchin, 1985), children’s characteristics during school transition, e.g., children’s school liking, may have an impact to the relations of coparenting and parental involvement. A successful transition to school includes academic performance, i.e., language and early math, as well as emotional adjustment, i.e., school liking (Buhs and Ladd, 2001). School liking, defined as the extent to which children profess to like or dislike school (Ladd et al., 1996), is positively associated with young children’s school performance, including behavioral engagement, e.g., cooperative classroom behavior, and achievement, e.g., cognitive skills (Ladd et al., 2000). When children exhibit low levels of school liking, parents may increase their involvement at home and in school to assist their children’s adjustment (Tao et al., 2019). Given that supportive coparenting is positively associated with parental involvement, parents of children with low levels of school liking may exhibit more parental involvement than those of children with high levels of school liking, because they want to support their children’s adjustment. Similarly, children’s school liking may buffer the negatively association between conflicted coparenting and parental involvement. Figure 1 displays the moderation plot. Parents of children with low levels of school liking may increase their involvement when the level of conflicted coparenting is high. However, such moderating effects have not been examined.

### Gender Difference and Chinese Context

Mothers’ and fathers’ differential gendered role within the context of coparenting is likely to influence their parental involvement. Specifically, the link between supportive coparenting and home-based involvement was found to be significantly stronger for mothers, and the link between supportive coparenting and school-based involvement was significantly stronger for fathers (Berryhill, 2017). The underlying mechanism may line up with the gender distinctions of specific aspects of family life (Allen and Hawkins, 1999). As mothers typically adopt the cultural norm of maintaining the home and involved more in childcare tasks, increased coparenting support may reinforce mothers’ cultural identity and the role they play as the center of care in family life, and this in turn may extend to greater levels of home involvement (Shumow and Lomax, 2002). By contrast, father involvement is highly influenced by ecological forces such as coparenting dynamics (Varga et al., 2014; Lau and Power, 2018), and fathers are generally less involved in school events than mothers (Tan and Goldberg, 2009). Fathers, therefore, may rely more on the guidance and support from mothers, i.e., coparenting, to communicate and coordinate the child’s school activities. Finding of these studies suggests that both parents should be involved in coparenting and parental involvement study to understand the gendered influence on these relations.

The associations among coparenting, child school liking, and parental involvement may vary across cultures, as parents from different countries may value children’s school liking differently (Seginer and Vermulst, 2002). In China, children’s school liking may strongly influence parental involvement because Chinese parents have long been identified as having high expectations for children’s academic achievement and be comparatively highly involved in school transition (Chan, 2010; Cheung and Pomerantz, 2011; Lau, 2014). Now, traditional gendered parenting roles, such as the caregiving mother and the working father, are slowly breaking down (Liu et al., 2016). At the same time, contemporary Chinese fathers are eager to support their children’s development (Lau, 2016). With these two facts in mind, we will investigate the associations between coparenting experiences and parental involvement during school transitions in a Chinese context and examine how children’s school liking moderates such associations.

### This Study

The longitudinal relation between coparenting and parental involvement, and the role of children’s school liking in moderating this relation, has not been fully explored. Given that theoretical reviews and empirical studies differ in their underlying conceptualizations of coparenting (e.g., Margolin et al., 2001; Feinberg, 2003), *cooperation* (i.e., the extent parents support and respect each other as parents) was adopted in this study as the major construct of supportive coparenting as it is widely used in previous studies (e.g., Lau and Power, 2019), and *triangulation* (i.e., parents involve the child in parental conflict) were adopted as the major construct of conflicted coparenting because it is more related to parent-child relations. The purpose of this study was to examine the longitudinal associations between coparenting experience and parental involvement in transition to primary school, and the moderation role of children’s school liking in a Chinese context. Based on the spill over hypothesis, we hypothesized that:

1. Cooperation would be positively associated with parental involvement. Triangulation would be negatively associated with parental involvement.
2. The positive association between cooperation and parental involvement would be more robust for parents of children with low levels of school liking.
3. The negative association between triangulation and parental involvement would be buffered by children’s low levels of school liking.

## Materials and Methods

### Sample

Stratified random sampling was used to recruit 10 kindergartens in each of the three strata (i.e., high, middle, and low income) developed based on the median monthly household incomes of the districts (Hong Kong Census and Statistics Department, 2012). Invitation letters were sent to kindergartens, and phone calls were made to the principals. Ten kindergartens agreed to participate in the study: three from the high-income stratum, four from the middle-income stratum, and three from low-income stratum. Parents of 324 of 621 children consented to participate. At T1, children-female=51% were 70.57months old on average, with an *SD*=3.70months. The median age range of mothers and fathers was 31–40years, and the median education level for both mothers and fathers was secondary education. The median range of monthly household income was HK$30,001–40,000 (US$ 3,861–5,148), similar to the median monthly household income of Hong Kong families of HK$24,890 (Hong Kong Census and Statistics Department, 2012). At T2, parents of 252 children (female=52%) completed the study. Attrition rates (22.2%) were considered acceptable due to difficulty retaining families when children changed schools. One-way ANOVA showed that families who dropped out had significantly lower mother and father education levels and lower levels of father-reported maternal cooperation.

### Procedure

A two-wave longitudinal design was adopted in this study. Time 1 (T1) data were collected in the final year of kindergarten and Time 2 (T2) data were collected in the first year of primary school. The interval between two-time points was 10months. Parents completed questionnaires and received tokens of appreciation for their participation at each time point. They reported the spouse’s coparenting behavior at T1, and their own parental involvement at T1 and T2. Children’s school liking was measured by puppet interview in the kindergarten at T1.

### Measurements

Coparenting experience was measured by using two of the three subscales of the Coparenting Questionnaire developed by Margolin et al., 2001. The cooperation subscale includes five items such as “My spouse tells me lots of things about this child.” The triangulation subscale includes four items such as “My spouse uses this child to get back at me.” Parents rated their spouse’s coparenting behavior on a five-point Likert scale with 1=never, 5=always. Higher scores mean higher levels of perceived cooperation/triangulation from the spouse. These subscales have been shown to be reliable in previous Chinese samples (Lau, 2017) and in this study (*α* ranged from 0.73 to 0.86 for mothers and fathers).

Parental involvement behavior was assessed using the 26-item Chinese Early Parental Involvement Scale (CEPIS) developed by Lau et al. (2011). The CEPIS includes two dimensions and six subdimensions that capture the multidimensional nature of Chinese parental involvement during the early childhood years (Home involvement: parent instruction, parent discussion, language and cognitive activities, and homework involvement; School involvement: home-school conferencing and preschool/school involvement). Parents rated their involvement on a five-point Likert scale (1=highly inaccurate; 5=highly accurate). The score of home and school involvement was computed by averaging the raw score of each dimension’s items. A higher score means higher levels of involvement. These subscales have been shown to be reliable in previous Chinese samples (Lau and Power, 2018) and in this study (*α* ranged from 0.78 to 0.91 for mothers and fathers).

Children’s school liking was measured using puppet interviews in their kindergarten. The six school liking items, such as “Are you happy when you are at school?,” were derived from the School Liking and Avoidance Questionnaire (Ladd and Price, 1987). In the interview, two opposite statements were presented by two identical hand puppets. One puppet says, “I am happy when I am at school,” another then says “I am not happy when I am at school.” Children were asked which puppet was most like them and indicate whether they were “very much alike” or only “slightly alike.” The response was recorded on a four-point scale, from 1=very much alike the negative statement to 4=very much alike the positive statement. Higher scores mean higher levels of school liking. The scale has been shown to be reliable in the present study (*α*=0.83).

### Analytic Plan

Data analysis was conducted using SPSS AMOS, version 23 (Arbuckle, 2017). Missing data were handled by using the Full Information Maximizing-Likelihood (FIML) method. Preliminary analyses were conducted to determine the descriptive characteristics and correlations among measured variables across time points. Path analysis models were applied to examine the moderation effect of children’s school liking on the longitudinal associations between coparenting experience and parental involvement. T1 coparenting experience, i.e., cooperation and triangulation, T1 children’s school liking, and the interactional variables of coparenting experience and children’s school liking were set as independent variables (IV). T2 parental involvement, i.e., home and school involvement, was set as dependent variable (DV), controlled by T1 parental involvement. The analyses were conducted separately for mothers and fathers. To avoid multicollinearity, all IVs constituting interaction terms were centered (Aiken, 1991). Model fit was assessed using the chi square statistic/df (<3.0), the CFI (≥0.95), and the RMSEA (≤0.08; Hooper et al., 2008). Moderation effect was determined by the significant level of the interactional terms less than 0.05. If a significant interactional effect was detected, a simple slope test was conducted using SPSS PROCESS (Hayes, 2012).

## Results

Table 1 shows the valid cases (*N*), means (*M*), SD, and correlations among the measured variables at both time points. For all the measured variables, skewness was less than 3, and kurtosis was less than 8, indicating that the non-normality of the data was not a problem. Correlation analyses showed that maternal cooperation at T1 was positively associated with paternal involvement at home and school at T2, while paternal cooperation at T1 was positively associated with maternal involvement at home at T2. Maternal and paternal triangulation at T1 was not associated with spousal parental involvement at T2.

**Table 1:** Valid cases (*N*), means (*M*), SD, and correlations among key variables.

	*N*	*M*	*SD*	1	2	3	4	5	6	7	8	9	10	11	12	13
1. M cooperation (T1)	311	3.23	0.81	-												
2. M triangulation (T1)	311	1.31	0.45	−0.21^**^	-											
3. F cooperation (T1)	306	3.80	0.71	0.37^**^	−0.22^**^	-										
4. F triangulation (T1)	306	1.50	0.67	−0.19^**^	0.41^**^	−0.20^**^	-									
5. M school involvement (T1)	319	3.05	0.77	0.17^**^	0.03	0.21^**^	0.05	-								
6. M home involvement (T1)	319	3.97	0.52	0.32^**^	−0.16^**^	0.29^**^	−0.06	0.60^**^	-							
7. M school involvement (T2)	237	3.21	0.67	0.13	0.06	0.12	−0.03	0.54^**^	0.29^**^	-						
8. M home involvement (T2)	237	4.05	0.48	0.27^**^	−0.14^**^	0.24^**^	−0.06	0.41^**^	0.63^**^	0.50^**^	-					
9. F school involvement (T1)	308	2.33	0.82	0.24^**^	−0.03	0.17^**^	−0.03	0.19^**^	0.09	0.00	0.06	-				
10. F home involvement (T1)	308	3.50	0.58	0.47^**^	−0.11	0.36^**^	0.02	0.19^**^	0.25^**^	−0.02	0.12	0.62^**^	-			
11. F school involvement (T2)	209	2.47	0.81	0.22^**^	−0.02	−0.00	−0.04	0.09	0.04	0.13	0.14^**^	0.50^**^	0.39^**^	-		
12. F home involvement (T2)	209	3.62	0.54	0.33^**^	−0.12	0.20^**^	0.01	0.02	0.05	0.12	0.20^**^	0.28^**^	0.47^**^	0.49^**^	-	
13. Child School liking (T1)	324	3.69	0.43	0.06	−0.16^**^	0.10	−0.03	0.07	0.07	0.01	0.08	0.01	0.02	−0.05	0.01	-

M, mother; F, father; T1, time 1; and T2, time 2.

** *p*<0.01.

The model fit for all path models was satisfactory [all chi square statistic/df (<3.0), the CFI (≥0.95), and the RMSEA (≤0.08)]. Table 2 displays the moderating effects of children’s school liking on coparenting and parental involvement. In the mother model, the interaction between cooperation and children’s school liking was significant for subsequent school involvement (*B*=−0.08, *p*=0.049) but not for home involvement (*B*=−0.04, *p*=0.11). A simple slope test revealed that the positive association between perceived cooperation and school involvement was only significant for mothers of children with high levels of school liking (i.e., 1 SD above the mean, *p*<0.05). This implies that children’s school liking significantly moderated the relations between mother perceived cooperation and school involvement. Mothers of children with high levels of school liking were involved more in school at T2 when they perceived more cooperation from the spouse at T1.

**Table 2:** The moderating effects of children’s school liking on coparenting and parental involvement.

	DV=M home involvement (T2)			DV=M school involvement (T2)		
	*B*	*SE*	*p*	*B*	*SE*	*p*
Cooperation	0.06	0.03	0.07	0.04	0.05	0.43
Child school liking	0.03	0.06	0.60	−0.08	0.10	0.38
Cooperation * Child school liking	−0.04	0.02	0.11	−0.08	0.04	0.049
M home involvement (T1)	0.63	0.05	<0.001	-	-	-
M school involvement (T1)	-	-	-	0.50	0.05	<0.001
M education	0.04	0.02	0.03	0.02	0.03	0.51
Family income	−0.01	0.02	0.80	−0.07	0.05	0.02
Triangulation	−0.08	0.05	0.14	0.06	09	0.50
Child school liking	0.02	0.06	0.78	−0.05	0.10	0.61
Triangulation * Child school liking	0.02	0.02	0.27	0.01	0.03	0.68
M home involvement (T1)	0.65	0.04	<0.001	-	-	-
M school involvement (T1)	-	-	-	0.50	0.05	<0.001
M education	0.05	0.02	0.04	0.02	0.03	0.55
Family income	−0.00	0.02	0.83	−0.07	0.03	0.02
	**DV=F home involvement (T2)**			**DV=F school involvement (T2)**		
Cooperation	0.07	0.05	0.19	−0.08	0.07	0.25
Child school liking	0.01	0.08	0.88	0.00	0.12	0.99
Cooperation * Child school liking	−0.03	0.03	0.37	−0.11	0.05	0.02
F home involvement (T1)	0.46	0.06	<0.001	-	-	-
F school involvement (T1)	-	-	-	0.47	0.06	<0.001
F education	0.03	0.03	0.21	0.05	0.04	0.22
Family income	0.00	0.03	0.93	−0.01	0.04	0.85
Triangulation	−0.01	0.05	0.78	−0.05	0.08	0.54
Child school likin	0.06	0.08	0.42	0.02	0.12	0.86
Triangulation * Child school likin	0.13	0.03	<0.001	0.12	0.04	0.002
F home involvement (T1)	0.48	0.06	<0.001	-	-	-
F school involvement (T1)	-	-	-	0.47	0.06	<0.001
F education	0.03	0.03	0.21	0.05	0.04	0.25
Family income	0.00	0.03	0.93	−0.01	0.04	0.90

M, mother; F, father; T1, time 1; and T2, time 2.

For fathers, the interaction between cooperation and children’s school liking was significant for subsequent school involvement (*B*=−0.11, *p*=0.02) but not for home involvement (*B*=−0.03, *p*=0.37). A simple slope test revealed that the negative association between father perceived cooperation and school involvement was only significant for fathers of children with low levels of school liking (i.e., 1 SD below the mean, *p*<0.05). Further, the interaction between triangulation and children’s school liking was significant for both subsequent home involvement (*B*=0.13, *p*<0.001) and school involvement (*B*=0.12, *p*=0.002). Specifically, the positive association between father-perceived triangulation and school involvement was only significant for fathers of children with low school liking (*p*<0.05). Fathers of children with high levels of school liking decreased their home involvement when they perceived more triangulation (*p*<0.05), while fathers of children with low levels of school liking increased their home involvement when they perceived more triangulation (*p*<0.05).

## Discussion

This study investigated the longitudinal associations between coparenting experience and parental involvement during the transition to primary school in a Chinese context, and the moderation role of children’s school liking in kindergarten in these associations. Partially consistent with the hypothesis, maternal cooperation was positively associated with paternal involvement at home and in school, and paternal cooperation was positively associated with maternal involvement at home. Children’s school liking in kindergarten moderated the longitudinal associations between coparenting experience and parental involvement. When perceived more cooperation, mothers of children with high levels of school liking became more involved in school, while fathers of children with low levels of school liking were less involved in school at T2. When perceived more triangulation, fathers of children with high levels of school liking became less involved at home, and fathers of children with low levels of school liking involved more at home and school at T2.

Consistent with previous studies, higher levels of cooperation were associated with higher levels involvement for both mothers and fathers (e.g., Schoppe-Sullivan et al., 2008; Hohmann-Marriott, 2011; Berryhill, 2017). Extending the results of previous literature that measured only one aspect of/a general parental involvement, results of this study suggest that supportive spousal coparenting longitudinally predicted maternal involvement at home and paternal involvement at home and in school. A possible explanation is that mothers are generally more involved at home and that spouse’s supportive coparenting reinforces their role as the center of care in family life (Shumow and Lomax, 2002). Since fathers are generally less involved than mothers (Tan and Goldberg, 2009), supportive coparenting by mothers might encourage their involvement both at home and in school. In contrast, triangulation was not associated with subsequent parental involvement, which is at odds with previous study (Buckley and Schoppe-Sullivan, 2010; Jia and Schoppe-Sullivan, 2011). The discrepancy may be due to a comparatively narrow definition of “parental involvement” (i.e., in caregiving and play) in previous study. Considering a broader range of parental involvement (incl. at home and in school) may result in a different link with coparenting.

When perceived more cooperation, mothers of children with high levels of school liking showed more involvement in school, which are not consistent with our hypothesis. This may imply that children’s school liking is a significant moderator for cooperation and maternal school involvement. When mothers perceived more cooperation, their confidence in communicating with and participating in the school may be further strengthened by the child’s high levels of school liking. Children’s school liking was not a moderator in the positive associations between cooperation and maternal home involvement. A plausible explanation is that Chinese mothers were found to perform their mothering role at home regardless of the stressors they experience (Kwan et al., 2015). Therefore, the quality of maternal involvement at home may be less susceptible to the stressors such as children’s school liking (Lau and Power, 2018).

Regarding fathers, those of children with low levels of school liking exhibited less school involvement when they perceived more cooperation from mothers, which is inconsistent with the hypothesis. This may be because fathers no longer feel they need to be involved as they can rely on the child’s mother when they perceive more cooperation, and low school-liking children are less likely to invite their parents to join school activities (Overstreet et al., 2005; Freund et al., 2018). Consistent with the hypothesis and compensation theory, fathers who perceived more triangulations were more involved at home and in school when their children showed low levels of school liking. A possible explanation is that Chinese fathers were found to rely on mothers’ support but at the same time, were eager to be highly involved in family affairs (Lau and Power, 2018). When mothers did not cooperate with them (i.e., high triangulation), fathers became more involved both at home and in school to support their children to have a better transition experience. Consistent with the spillover theory and our hypothesis, fathers of children with high levels of school liking showed decreased involvement at home when they perceived more triangulation, perhaps because fathers transfer the negative affect from spousal subsystem to parent-child subsystem and feel fine to decrease their home involvement as their children seem to adjust well in the transition.

### Implications for Theory and Practice

The present study contributes to the literature by revealing the moderating role of children’s school liking in the longitudinal associations between coparenting experiences and parental involvement for Chinese parents during school transitions. Consistent with spillover and compensation theories, coparenting experience at the mother-father level influenced parental involvement at the parent-child level, with the association significantly moderated by children’s school liking. The findings of this study reveal that negative coparenting, i.e., triangulation, could also lead to positive parental functioning when considering children’s school liking as a moderator. This supplements the consensus that positive coparenting leads to improvements in parental functioning and negative coparenting increase the risk of functioning problems (Solmeyer and Feinberg, 2011; Lau and Power, 2018; Kanter and Proulx, 2019). As such, an important implication of this study would be to take children’s characteristics into consideration when enhancing positive coparenting and reducing negative coparenting to increase parental involvement during school transitions. When a child showed low levels of school liking, parents-especially fathers-perceiving more cooperation should be encouraged to communicate more with the school to aid the children’s adjustment. Meanwhile, mothers of children with high levels of school liking should be encouraged to decrease their triangulation to their spouse to improve paternal involvement at home. Fathers of children with low levels of school liking should be encouraged to keep their involvement level to support the child.

### Strengths and Limitations

This study has several strengths. First, although accumulating studies have suggested that father involvement should be considered as an important part of family process (McWayne et al., 2013; Kim and Hill, 2015), paternal involvement in the context of school transition is relatively rare (for a review, see Kim and Hill, 2015). This study included fathers as a coparent in the associations between coparenting experience and parental involvement, and further involved children’s characteristics, i.e., school liking, as a moderator in the above associations. Second, supplementing to previous cross-sectional associations between coparenting experience and parental involvement, the longitudinal data in the present study described the change of parental involvement in school transition predicted by coparenting experience and children’s school liking. Third, given that the traditional adult methods, such as parental reports of child behavior, have been criticized for bypassing children’s own voices (Lambert et al., 2013), children’s school liking was measured using puppet interviews to better reflect children’s feelings.

Although innovative, this study has several limitations. First, the generalizability of the finding may be influenced by recruiting participants in Hong Kong. Hong Kong is a special administration region in China, adhering to Chinese culture but deeply influenced by other cultures due to its colonist history. Future studies may consider conducting study in more Chinese cities. Second, parental involvement and coparenting were measured by parents’ self-reports. By only using parental reports, there may be an informant bias because parental reports are based on subjective judgment. Future research should consider collecting parents’ data using observational measures and spouse reports. Third, as parents who dropped out from the study had low education levels and low levels of father-reported maternal cooperation, the findings of this study may not be representative enough of those parents. Future studies should consider focusing on specific groups of parents such as those with low levels of education. Fourth, child school liking was measured at T1 only (kindergarten). Future research could examine if longitudinal data of school liking could help reveal the direction of effect on children liking school and mother involvement, and whether parental involvement in school influences children’ school liking in the first grade.

## Data Availability Statement

The original contributions presented in the study are included in the article/supplementary material, further inquiries can be directed to the corresponding author.

## Ethics Statement

The studies involving human participants were reviewed and approved by Human Research and Ethics Committee. Written informed consent to participate in this study was provided by the participants’ legal guardian/next of kin.

## Author Contributions

ST conducted the analysis and wrote the paper. EL designed the study and guided the project. All authors contributed to the article and approved the submitted version.

## Funding

This work was supported by the Research Grant Council (ECS 28401914).

## Conflict of Interest

The authors declare that the research was conducted in the absence of any commercial or financial relationships that could be construed as a potential conflict of interest.

## Publisher’s Note

All claims expressed in this article are solely those of the authors and do not necessarily represent those of their affiliated organizations, or those of the publisher, the editors and the reviewers. Any product that may be evaluated in this article, or claim that may be made by its manufacturer, is not guaranteed or endorsed by the publisher.
